# Comparison of mechanistic models in the initial rate enzymatic hydrolysis of AFEX-treated wheat straw

**DOI:** 10.1186/1754-6834-3-6

**Published:** 2010-03-23

**Authors:** Russell F Brown, Frank K Agbogbo, Mark T Holtzapple

**Affiliations:** 1 HSB Solomon Associates LLC, 13455 Noel Road, Ste 1500, Dallas, TX 75240, USA; 2 Mascoma Corporation, 67 Etna Road, Suite 300, Lebanon, NH 03766, USA; 3 Department of Chemical Engineering, Texas A&M University, College Station, TX 77843-3122, USA

## Abstract

**Background:**

Different mechanistic models have been used in the literature to describe the enzymatic hydrolysis of pretreated biomass. Although these different models have been applied to different substrates, most of these mechanistic models fit into two- and three-parameter mechanistic models. The purpose of this study is to compare the models and determine the activation energy and the enthalpy of adsorption of *Trichoderma reesei *enzymes on ammonia fibre explosion (AFEX)-treated wheat straw. Experimental enzymatic hydrolysis data from AFEX-treated wheat straw were modelled with two- and three-parameter mechanistic models from the literature. In order to discriminate between the models, initial rate data at 49°C were subjected to statistical analysis (analysis of variance and scatter plots).

**Results:**

For three-parameter models, the HCH-1 model best fitted the experimental data; for two-parameter models Michaelis-Menten (M-M) best fitted the experimental data. All the three-parameter models fitted the data better than the two-parameter models. The best three models at 49°C (HCH-1, Huang and M-M) were compared using initial rate data at three temperatures (35°, 42° and 49°C). The HCH-1 model provided the best fit based on the F values, the scatter plot and the residual sum of squares. Also, its kinetic parameters were linear in Arrhenius/van't Hoff's plots, unlike the other models. The activation energy (*Ea*) is 47.6 kJ/mol and the enthalpy change of adsorption (Δ*H*) is -118 kJ/mol for *T. reesei *enzymes on AFEX-treated wheat straw.

**Conclusion:**

Among the two-parameter models, Michaelis-Menten model provided the best fit compared to models proposed by Humphrey and Wald. For the three-parameter models, HCH-1 provided the best fit because the model includes a fractional coverage parameter (ϕ) which accounts for the number of reactive sites covered by the enzymes.

## Background

Over the years, two kinds of cellulose hydrolysis models have been developed: empirical and mechanistic models. As empirical models lack a firm theoretical foundation, it is impossible to extend them beyond the range of data to which they were fit. Therefore, our attention was focused on mechanistic models that describe the reaction mechanism between lignocellulosic biomass and enzyme. In order to formulate an appropriate mechanistic model, we needed to know how enzymes hydrolyze lignocellulosic substrates.

The hydrolysis of lignocellulosic substrates depends on enzyme characteristics [1], including: (1) adsorption of enzyme onto lignocellulosic biomass prior to reaction; (2) end-product inhibition which is competitive [2] or noncompetitive [3]; (3) synergy of the various enzyme components; and (4) mass transfer limitations affecting the transport of the enzyme to the substrate [1]. Enzymatic hydrolysis also depends on substrate characteristics including: (1) lignin distribution; (2) the presence of other components such as hemicellulose, proteins and fats; (3) particle size; and (4) crystallinity [4].

Incorporating all these factors into a single model is cumbersome and highly complicated. We, therefore, divided these factors into short-term and long-term factors. For short-term hydrolysis (initial rate), Fan and Lee [5] have shown that: (1) product inhibition is not important; (2) hydrolysis is least affected by mass transfer effects; (3) chemical pretreatment is important; and (4) the pseudo-steady state assumption can be used [5]. For long-term hydrolysis, Fan and Lee [6] have indicated that: (1) rate is higher initially but changes later due to product inhibition; (2) pseudo-steady state assumptions do not apply; and (3) changes occur in the crystallinity index and surface area. Literature models consider the above factors and, in some cases, differential equations were used to model both the short-term and long-term hydrolysis process [7,8]. The simplest forms consider a single substrate and a single enzyme system.

Table 1 summarizes the cellulose hydrolysis models that have appeared in the literature. They can broadly be categorized as two- and three-parameter models [9]. The more complex mechanistic models consider multiple substrates (amorphous and crystalline cellulose) and the different enzyme components.

**Table 1:** Summary of models.

Model	Two-parameter models	References
2A		Ghose and Das [10], Dwivedi and Ghose [14], Howell and Stuck [11], Caminal *et al. *[15], Gan *et al. *[7]

2B		Humphrey *et al. *[16] Movagharnejad and Sohrabi [17]

2C		Wald *et al. *[18]
	Three-parameter models	

3A		Fan and Lee [20]

3B		Huang [21]

3C		Holtzapple *et al. *[22]

### Model 2A

The Michaelis-Menten (M-M) model was used to describe the hydrolysis of Solka Floc and avicel [10-13]. The hydrolysis of alkali-treated bagasse by *Trichoderma reesei *cellulase was evaluated using M-M kinetics with competitive inhibition [14]. The M-M model was used by Caminal *et al*. [15], but the authors could not distinguish between competitive and noncompetitive inhibition by cellobiose. The M-M model works on the assumption that the substrate concentration is much higher than the enzyme concentration and this may not always be the case. A mechanistic model similar to M-M kinetics was proposed and differential equations were solved for the different substrate components [7].

### Model 2B

The shrinking-site hydrolysis model with a Langmuir-type adsorption isotherm was used in order to get three different rate equations for cellulose, cellobiose and glucose [16]. Recently, the shrinking-site model was extended to rice pollards, sawdust, wood particles and used paper [17].

### Model 2C

The model has a similar mathematical form to M-M, except that an enzyme term appears in the denominator rather than a substrate term [18,19].

### Model 3A

A mechanistic model proposed by Fan and Lee that describes the hydrolysis of cellulose and cellobiose, but does not include an adsorption step [20].

### Model 3B

This model was proposed by Huang when cellulose hydrolysis by *T. viride *cellulase was modelled using the M-M mechanism with competitive inhibition [21].

### Model 3C

The HCH-1 model was proposed by Holtzapple *et al. *[22], which is essentially the M-M mechanism with noncompetitive inhibition and a parameter to account for the number of reactive sites covered by the enzymes. A pseudo-steady state approximation for the HCH-1 model was developed [23] and recently applied to lime pretreated corn stover [24].

Most of the mechanistic models used to describe cellulose hydrolysis in the literature fit into the six mathematical forms presented in Table 1[9]. In some cases, the constants are interpreted differently. In other cases, the models are applied multiple times to each enzyme and substrate component. It is worthwhile to compare these models in order to determine their relative merits. To simplify the system, an initial rate data was generated from ammonia fiber explosion (AFEX)-treated wheat straw that was hydrolyzed with *T. reesei* cellulase. The data were fitted to the various models so they could be compared on an equal basis.

## Results and discussion

**Figure 1:**
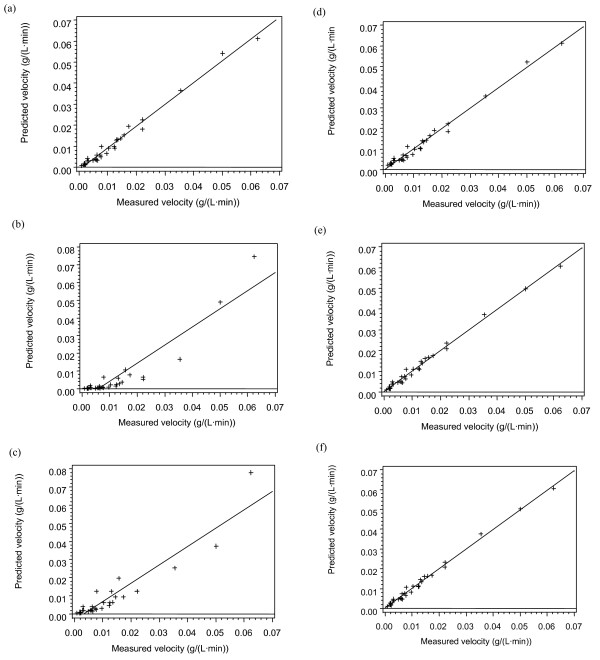
**Scatter plots at 49°C**. (a) Model 2A (Michaelis-Menten); (b) Model 2B (Humphrey); (c) Model 2C (Wald); (d) Model 3A (Fan and Lee); (e) Model 3B (Huang); (f) Model 3C (HCH-1).

**Figure 2:**
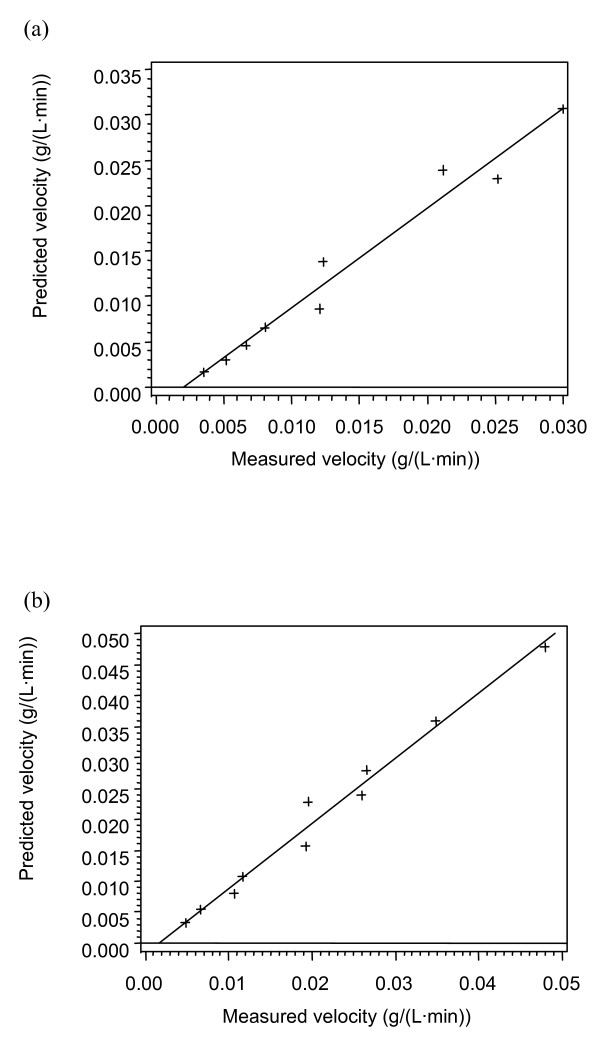
**Scatter plot for Model 2A (Michaelis-Menten)**. (a) 35°C; (b) 42°C.

**Figure 3:**
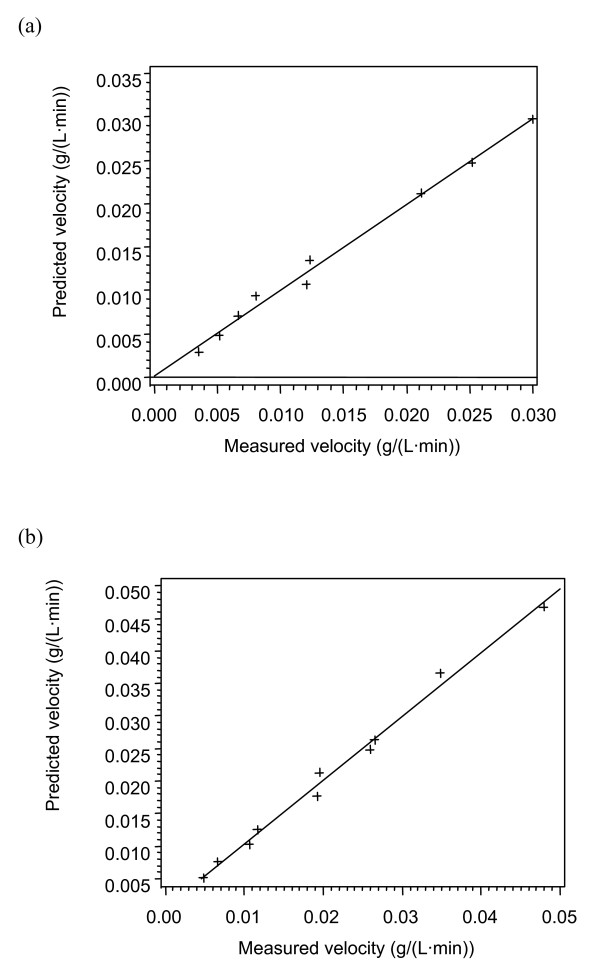
**Scatter plot for Model 3B (Huang)**. (a) 35°C (b) 42°C.

**Figure 4:**
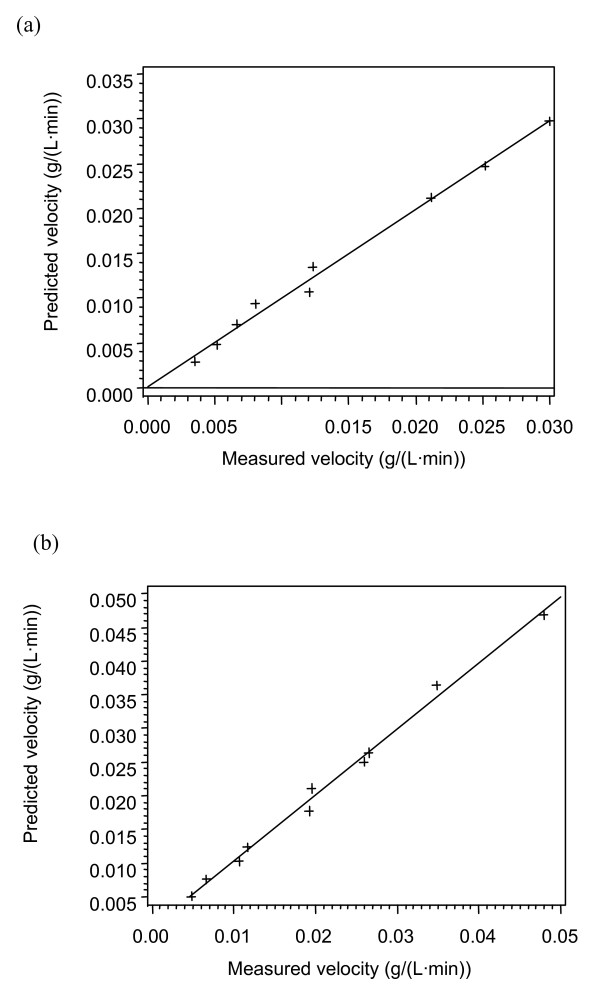
**Scatter plot for Model 3C (HCH-1)**. (a) 35°C; (b) 42°C.

The enzyme loadings and substrate conditions at the specific temperatures are given in Tables 2, 3 and 4. The data (Tables 1, 2, 3, 4) were subjected to statistical analysis (analysis of variance and scatter plots). For each model, a plot of the predicted velocity versus the measured velocity (scatter) was made (Figures 1, 2, 3 and 4). The *F *values and model parameter estimates were obtained for each model (Tables 5, 6 and 7). The plots together with the *F *values were used to compare the models.

**Table 2:** Initial velocity data for enzymatic hydrolysis of ammonia fibre explosion (AFEX)-treated wheat straw at 49°C.

Experiment. No.	[*S*] (g/L)	[*E*] (g/L)	*r*_s _× 100 (g/L·min)
1	15.60	0.738	0.629*

2	3.85	1.145	0.648

3	1.93	0.736	0.194

4	3.85	0.364	0.220

5	7.90	0.365	0.197

6	7.90	0.735	0.580

7	47.77	0.739	0.792

8	3.91	1.138	0.485

9	7.86	1.138	0.739

10	15.52	1.138	1.257

11	15.49	0.365	0.297

12	31.01	0.365	0.310

13	15.47	0.187	0.215

14	15.47	0.094	0.098

15	15.50	0.364	0.323

16	7.76	0.364	0.216

17	15.43	1.712	1.460

18	3.87	1.712	0.770

19	15.41	1.141	1.044

20	31.01	1.141	1.319

21	7.76	0.730	0.616

22	15.43	2.287	2.229

23	3.89	2.287	0.979

24	48.73	1.138	1.579

25	3.97	4.559	1.358

26	15.72	4.561	3.549

27	48.34	4.561	6.241

28	7.74	4.561	2.218

29	3.93	3.412	1.237

30	3.89	6.825	1.738

31	15.50	6.825	5.014

* *r*_s _= 0.00629 g/(L·min)

**Table 3:** Initial velocity data for enzymatic hydrolysis of ammonia fibre explosion (AFEX)-treated wheat straw at 42°C.

Experiment No.	[*S*] (g/L)	[*E*] (g/L)	*r*_s _× 100 (g/L·min)
1	48.34	3.412	3.479*

2	15.50	1.142	1.063

3	31.01	1.153	1.171

4	3.93	1.153	0.483

5	15.39	3.420	2.598

6	48.15	4.562	4.795

7	8.49	4.562	1.956

8	7.74	1.152	0.666

9	11.92	4.563	2.653

10	11.90	2.548	1.927

* *r*_s _= 0.03479 g/(L·min)

**Table 4:** Initial velocity data for enzymatic hydrolysis of ammonia fibre explosion (AFEX)-treated wheat straw at 35°C.

Experiment No.	[*S*] (g/L)	[*E*] (g/L)	*r*_s _× 100 (g/L·min)
1	48.34	3.412	2.516*

2	15.50	1.144	0.668

3	30.82	1.153	0.804

4	3.89	1.153	0.357

5	15.49	3.420	1.232

6	48.15	4.562	2.998

7	7.74	1.152	0.515

8	25.42	4.563	2.117

9	16.10	2.092	1.209

* *r*_s _= 0.02516 g/(L·min)

**Table 5:** Parameter estimates at 49°C.

Models	Parameter 1	Parameter 2	Parameter 3	*F *value	Residual sum of square
2A	*k *= 0.0200 (g/(g·min))	*K*_m _= 23.5237 (g/L)	-	1311.34	0.000117

2B	*K *= -0.00042 (L/g)^(1/3)^·min^-1^	α = -9.1015 (g/L)	-	60.26	0.00208

2C	*k *= 8489674 (g/L)	α = 2.4E10 (g/(g·min))	-	245.34	0.00117

3A	*k *= 0.00156 (g/(L·min))	κ = 0.0204 (g/(g·min))	α = 27.1162 (g/L)	1072.28	0.000076

3B	κ = 0.0190 (g/(g·min))	α = 12.7035 (g/L)	ε = 1.7855 (g/g)	2219.86	0.000045

3C	κ = 0.0168 (g/(g·min))	α = 10.2269 (g/L)	ε = 2.4631 (g/g)	2232.79	0.000045

**Table 6:** Parameter estimates at 42°C.

Models	Parameter 1	Parameter 2	Parameter 3	*F *value	Residual sum of square
2A	*k *= 0.0137 (g/(g·min))	*K*_m _= 14.7743 (g/L)	-	548.33	0.000043

3B	κ = 0.0138 (g/(g·min))	α = 5.5471 (g/L)	ε = 2.4092 (g/g)	1044.74	0.000013

3C	κ = 0.0115 (g/(g·min))	α = 3.5973 (g/L)	ε = 3.6250 (g/g)	3428.53	0.000004

**Table 7:** Parameter estimates at 35°C.

Models	Parameter 1	Parameter 2	Parameter 3	*F *value	Residual sum of squares
2A	*k *= 0.00978 (g/(g·min))	*K*_m _= 21.9288 g/L)	-	199.41	0.000042

3B	κ = 0.0119 (g/(g·min))	α = 5.7541 (g/L)	ε = 7.4772 (g/g)	805.81	0.000006

3C	κ = 0.00748 (g/(g·min))	α = 1.3730 (g/L)	ε = 8.2915 (g/g)	1196.19	0.000004

For the two-parameter models at 49°C, Model 2A (M-M) is clearly the best. The *F *values and the residual sum of squares (RSS) favour the M-M mechanism. Model 2B (Humphrey) produced negative parameters, so it is clearly inadequate. The fit from Model 2C (Wald) is very poor from the scatter plots. Of the three-parameter models, Model 3C (HCH-1) provided the best fit. The HCH-1 model has the highest *F *value of 2232 and provided a better fit from the scatter plot. Model 3B (Huang) has an *F *value of 2219 and the scatter plots were very similar to HCH-1. Therefore, Model 3B (Huang) is the closest competitor to the HCH-1 model.

As the *F *value, RSS or the correlation coefficient (*R*^2^) provide a comparison between models with the same number of parameters; they will be used to compare models with the same number of parameters [25-27]. Among the two-parameter models at 49°C, the *F *values and the RSS show that Model 2A is the best model. The two best models for the three-parameter models at 49°C are Model 3B and Model 3C based on the *F *values and the RSS. These three models (2A, 3B and 3C), were further tested at 35° and 42°C. Among the two three-parameter models tested at 35° and 42°C, the HCH-1 model (Model 3C) provided the best fit based on the *F *values and the RSS.

**Figure 5:**
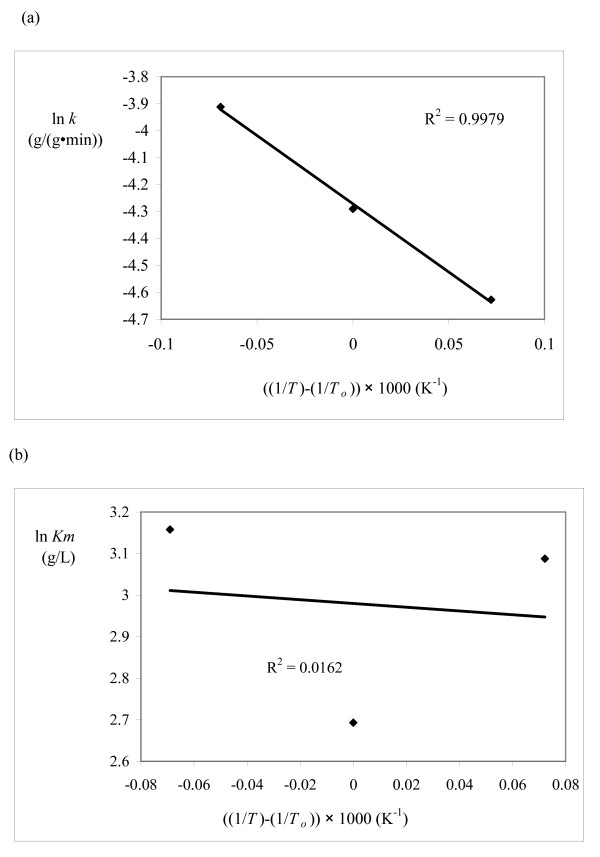
**Model 2A (Michaelis-Menten)**. Arrhenius/van't Hoff plots for (a) *k *and (b) *K*_*m*_.

**Figure 6:**
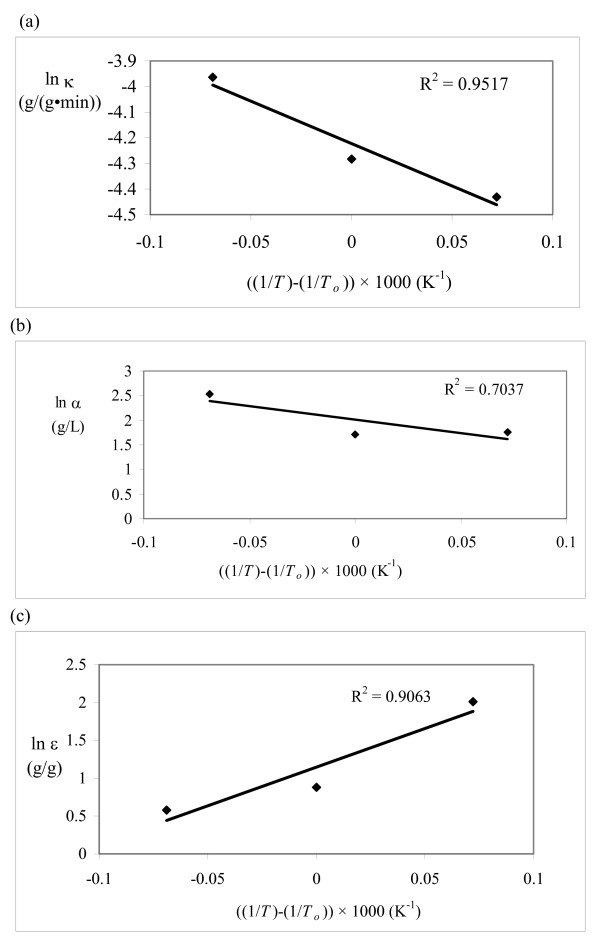
**Model 3B (Huang)**. Arrhenius/van't Hoff plots for (a)κ, (b) α and (c) ε.

**Figure 7:**
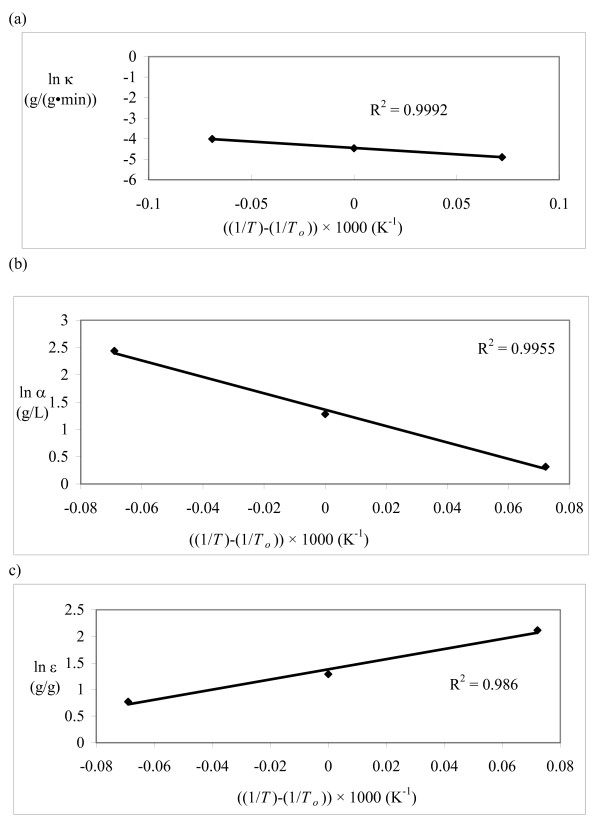
**Model 3C (HCH-1)**. Arrhenius/van't Hoff plots for (a) κ, (b) α and (c) ε.

For a kinetic model to be valid, the rate constant should follow the Arrhenius equation and the adsorption/desorption parameters should follow the van't Hoff equation. The kinetic parameters from this study were plotted on Arrhenius/van't Hoff plots (Figures 5, 6 and 7) using 315°K as the reference temperature (*T*_*o*_). The HCH-1 plot provided the best fit from the R^2 ^values (Figure 7a - c), therefore the rate constants follow the Arrhenius equation. The kinetic parameters in the HCH-1 model for AFEX-treated wheat straw are temperature dependent and can, therefore, be predicted by the Arrhenius/van't Hoff relationships. The coverage parameter (ε) depends on the adsorption parameter, which explains the van't Hoff dependence on temperature. HCH-1 provided the best fit as it has a fractional coverage parameter (ϕ) that accounts for the number of reactive sites covered by the enzyme.

The activation energy (*E*_*a*_) is 47.6 kJ/mol and the enthalpy change of adsorption (Δ*H*) is -118 kJ/mol for AFEX-treated wheat straw. The activation energy compares very well with previous work on cotton fibres, pulp and cellobiose (Table 8) [28-30]. Table 9 summarizes results from the comparison of the models. The HCH-1 model (Model 3C) is the only model that meets all the criteria specified in Table 9.

**Table 8:** Summary of cellulase activation energies and heats of adsorption.

Enzyme Source	Substrate	*Ea* (kJ/mol)	Δ*H *(kJ/mol)	Reference
*Trichoderma reesei*	AFEX-treated wheat straw	47.6	-118	This Work

*T. viride *(endo)	Cotton fibres	54.6	16.5	Beltrame *et al*. [28]

*T. viride *(endo)	Pulp	55.1	-48.9	Beltrame *et al*. [28]

*T. viride *(exo)	Cotton fibres	137.4	66.1	Beltrame *et al*. [28]

*T. viride *(exo)	Pulp	137.4	76.9	Beltrame *et al*. [28]

*T. viride*	Cellobiose	45.1	20.9	Beltrame *et al*. [28]

*Aspergillus niger*	Cellobiose	46.1	-	Calsavara *et al*. [29]

*T. reesei*	Avicel	29.8	-148	Drissen *et al*. [30]

AFEX, ammonia fibre explosion

**Table 9:** Summary of model comparison results.

Model	*F *value	Parameter estimates*	Scatter plots	Arrhenius plots
2A	√	√	√	X

2B	X	X	X	

2C	X	√	X	

3A	X	√	√	

3B	√	√	√	X

3C	√	√	√	√

√ = Model success.

X = Model failure.

* Parameter estimates fail when estimates were negative.

## Conclusions

Among the two-parameter models, Model 2A (M-M) is the best, although it does not include an adsorption step prior to hydrolysis. Model 2B (Humphrey) introduced an adsorption parameter, a lumped constant which might be responsible for the negative parameters that were generated. Model 2C (Wald) and Model 3A (Fan and Lee) are based on a complex reaction system that did not adequately describe the data. Model 3B (Huang) assumed fast adsorption and slow reaction. It was good at a given temperature. However, there was more scatter in the Arrhenius plot compared to HCH-1. Model 3C (HCH-1) includes the fractional coverage parameter (ϕ) which accounts for the number of reactive sites covered by the enzyme. The inclusion of the coverage parameter gives HCH-1 a better fit for the data. At a fixed temperature, Model 3C (HCH-1) was comparable to Model 3B (Huang). However, Model 3C had much less scatter in the Arrhenius plot.

## Methods

### Pretreatment

Using the AFEX process [31], moist wheat straw was contacted with liquid ammonia. After thorough mixing, ammonia (which disrupts hydrogen bonds in cellulose) was instantaneously released to the atmosphere. This sudden decrease in pressure caused the liquid ammonia trapped in the cellulose fibres to 'explode', which decreased the crystallinity of the cellulose and increased the surface area.

In order to pretreat the wheat straw used in this study, 1370 g of ground wheat straw (0.08 g water/g dry biomass) was mixed with 142 mL of water to bring the moisture content to 0.19 g water/g dry biomass. The wheat straw was placed in an airtight container in an incubator at 35°C for at least 15 min in order to distribute the moisture evenly throughout the straw. Batches of 150 - 250 g of moist wheat straw were treated with ammonia at a ratio of 1.2 g NH_3_/g dry wheat straw in an AFEX apparatus [32] at 220 psig (1.62 MPa) and 125°F (52°C) for 15 min.

After this first treatment, all of the batches were recombined and allowed to dry for 36 h. Prior to the next treatment, the wheat straw was mixed with water to bring the moisture content to 0.20 g water/g dry biomass and the AFEX process was repeated. This procedure was repeated again, so that the entire amount of wheat straw was AFEX-treated a total of three times.

After treatment, the final moisture content was 0.18 g water/g dry biomass. In order to prevent changes in cellulose structure during storage, the treated wheat straw was kept frozen until its use in the hydrolysis runs. Table 10 lists wheat straw composition as measured by the forage fibre analysis of Goering and Van Soest [33], particle size analysis [3] and other physical properties.

**Table 10:** Physical properties of pretreated wheat straw.

No. of treatments	3
Moisture content (g H_2_O/g dry matter)	0.18

Cellulose (%)	36.9
Hemicellulose (%)	27.9
Lignin (%)	10.7
Cell solubles (%)	19.9
Protein (%)	2.3
Ash (%)	2.2

Average length (mm)	2.9 ± 0.9
Average width (mm)	0.8 ± 0.3

### Hydrolysis apparatus

**Figure 8:**
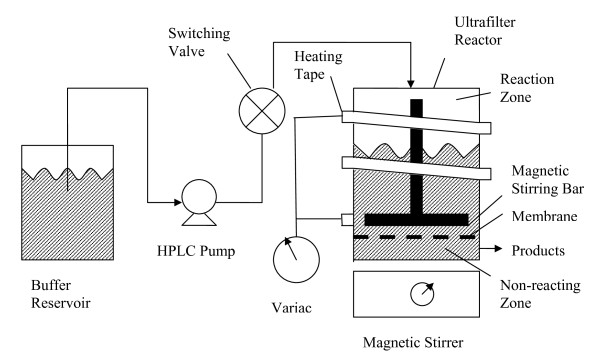
**Amicon filter apparatus**.

The enzymatic hydrolysis experiments were conducted in an apparatus employing an Amicon ultra-filter membrane (Figure 8). In order to perform the hydrolysis, the AFEX-treated wheat straw was placed in the Amicon stirred cell (10,000 MW-cutoff membrane filter) with 0.05 M, pH 4.8 citric acid buffer. The stirred cell was completely filled with solution. The apparatus was wrapped by a heating tape and the temperature was manually regulated using a Variac. When the desired temperature was achieved, insulation (polyurethane) was placed around the holder in order to maintain the temperature. The temperature could be maintained to within 0.1°C of the desired setting by adjusting the Variac setting or moving the insulation. In order to initiate the reaction, cellulase was injected into the Amicon filter holder using a six-port Rheodyne model 7125 high-performance liquid chromatograph (HPLC) switching valve with a 5-mL sample loop. The 10,000 MW-cutoff filter (Millipore PTGC 076 10) retained the AFEX-treated wheat straw and cellulase but allowed product (cellobiose and glucose) to pass.

### Enzymes

The enzymes used in this study were *T. reesei *cellulase (Genencor 300P) and β-glucosidase (Novozyme 188). The Novozyme 188, with a reported activity of 250 cellobiose units per gram, was purchased in liquid form and was kept refrigerated until use. As purchased, the Novozyme 188 contained about 40 g/L of glucose.

In order to remove the glucose in the Novozyme 188 by dialysis, an Amicon filter unit with a 10,000 MW cut-off filter was used. Two grams of the dialyzed Novozyme 188 was diluted with 0.05 M, 4.80 pH citrate buffer solution to bring the total volume to 1L. It was preserved with 0.03 wt% NaN_3_. This procedure reduced the glucose by 1000 times; the final diluted Novozyme 188 solution contained 0.04 g/L glucose. The β-glucosidase was added to each sample to convert cellobiose to glucose. The standard procedure was to add 100 μL of the diluted Novozyme 188 solution to the sample (0.5 - 1.0 mL) and incubate the sample at 50°C for 24 h. The concentrations of the glucose before and after β-glucosidase was added, were determined with YSI Model 27 glucose analyser. The glucose concentration before and after β-glucosidase addition was used to determine the cellobiose produced after hydrolysis.

### Data analysis

The ultra-filter (UF) cell was partitioned into two parts. The first compartment had a volume of 440 mL, which is where the reaction occurred. The second compartment, with a volume of 2 mL, was the space below the membrane where the effluent collected and was directed into the tube exiting the reactor. The cell was modelled as two perfectly mixed vessels in series. The glucose produced 30 min after reaction initiation was assumed to be the initial rate. The sugars present (glucose and cellobiose) inhibit the reaction. Glucose and cellobiose inhibition parameters determined by Cognata [34] and Holtzapple *et al*. [35] were used to correct the initial rates. As the sugar concentrations were small, little correction was required.

### Statistical analysis

The nonlinear regression procedure NLIN was used for the SAS programming. The Marquardt method was used for the iteration and the Hougaard option was used to determine the skewness. The analysis of variance tables provided information on the sum of squares, *F *values, model parameter estimates and skewness. Scatter plots indicated the goodness of fit. The best models for each temperature were determined and the kinetic parameters were fitted using Arrhenius/van't Hoff plots using the re-parameterized equations suggested by Kittrell [36]. For the experiments at 35° and 42°C, a sequential design of experiments was used to decrease the number of experiments required to determine the parameters [37].

## Abbreviations

AFEX: ammonia fibre explosion; M-M: Michaels-Menten model; RSSH: residual sum-of-squares under the null hypothesis for the lack of fit *F*-test; RSS: residual sum of squares; UF: ultra-filter; [*E*]: cellulase concentration; g/L, *k*: rate constant; g/(g·min), *K*_*m*_: Michaelis-Menten constant; g/L, *n*: total number of observations; *p*: difference in the number of parameters; *r*_*s*_: the rate of appearance of sugars; [*S*]: substrate concentration; g/L, *V*: rate of reaction; g/(L·min), α: lumped parameter; g/L, ε: coverage parameter; g/g, κ: rate constant; g/(g·min); ϕ: ratio of free substrate to total substrate, dimensionless.

## Competing interests

The authors declare that they have no competing interests.

## Authors' contributions

RFB carried out the experiments, performed data analysis and helped to draft the manuscript. FKA performed the data analysis and worked on writing the manuscript. MTH worked on the experimental design, coordination of the study and writing of the manuscript.
